# Inhibition/activation in bipolar disorder: validation of the Multidimensional Assessment of Thymic States scale (MAThyS)

**DOI:** 10.1186/1471-244X-13-79

**Published:** 2013-03-13

**Authors:** Chantal Henry, Amandine Luquiens, Christophe Lançon, Hélène Sapin, Marcel Zins-Ritter, Stephanie Gerard, Elena Perrin, Bruno Falissard, Michael Lukasiewicz

**Affiliations:** 1 INSERM, U995, IMRB, Department of Genetics, AP-HP, Henri Mondor-Albert Chenevier Group, Psychiatry, Univ Paris 12, Faculty of Medicine, IFR10, Créteil, France; 2 AP-HP, Paul-Brousse Hospital, Department of Psychiatry and Addictology, Villejuif, France; 3 Self-Perceived Health Assessment Research Unit, School of Medicine, La Timone University, Marseille, 13005, France; 4 Department of Psychiatry, Sainte-Marguerite University Hospital, Marseille, France; 5 Eli Lilly and Company, 24 Boulevard Vital Bouhot, Neuilly-Sur-Seine, 92200, France; 6 Private practice, Orvault, France; 7 INSERM, U669, Univ Paris-Sud and Univ Paris Descartes, UMR-S0669, Paris, France

**Keywords:** Emotional reactivity, Bipolar disorder, Factorial analysis, Scale, Clinical trial, Olanzapine

## Abstract

**Background:**

One of the major issues in clinical practice is the accurate differential diagnosis between mixed states and depression, often leading to inappropriate prescriptions of antidepressants in mixed states, and as a consequence, increasing the risk of manic switch and suicide. In order to better define the spectrum of mixed states, it may be useful to develop a dimensional approach. In this context, the MAThyS (Multidimensional Assessment of Thymic States) scale was built to assess activation/inhibition levels in all bipolar mood episodes, and to determine whether a clinical description in terms of activation/inhibition can help better define bipolar states with which both manic and depressive symptoms are associated. The aim of this paper is the validation of the MAThyS scale in 141 bipolar patients in acute states (manic, hypomanic, mixed, or depressive).

**Methods:**

The validation of the MAThyS scale was the primary outcome of this 24-week, phase III, open-label, olanzapine single-arm clinical trial. Principal component, factorial analysis, and Cronbach’s coefficient calculation (internal consistency) were performed. Concurrent validity (correlations with 17-item Hamilton Depression Rating Scale [HAMD-17], Hamilton Anxiety Rating Scale [HAMA], and Young Mania Rating Scale [YMRS]) and responsiveness to the clinical intervention were assessed (change in MAThyS scale and effect size) at 6 and 24 weeks.

**Results:**

Scree plot of eigenvalues identified a 2-dimension structure (“activation/inhibition level” and “emotional component”). Psychometric properties were good: Cronbach’s coefficient was >0.9. Concurrent validity was good with low correlation (−0.19) with the HAMA scale and a higher correlation at baseline with the YMRS (0.72) and HAMD-17(−0.43). As expected, the activation state was predominant in manic, hypomanic, and mixed states while inhibition was predominant in depressive states. MAThyS score improvement was observed (effect size: -0.3 at 6 and 24 weeks).

**Conclusions:**

The MAThyS demonstrated good psychometric properties. The MAThyS scale may help clinicians to better discriminate and follow bipolar episodes, especially the broad spectrum of mixed episodes.

**Trial registration:**

ClinicalTrials.gov registration identification number: NCT#002592722

## Background

The traditional categorical paradigm of the bipolar disorder spectrum (manic, depressive, and mixed states) has been recently completed by a dimensional paradigm with the description of a range of intermediate states (e.g., dysphoric mania and hypomania, mixed depression) which belong to a broad spectrum of mixed states [1,2]. One of the major issues in clinical practice is the accurate differential diagnosis between mixed states and depression, often leading to inappropriate prescriptions of antidepressants in mixed states, and as a consequence, increasing the risk of manic switch and suicide [3,4]. In order to better define the spectrum of mixed states, it may be useful to develop a dimensional approach. The dimensional paradigm classifies clinical conditions according to quantitative attributes rather than assignment to categories. This paradigm is particularly relevant when the attributes have no clear boundaries, as in the bipolar mood spectrum.

In this context, the MAThyS (Multidimensional Assessment of Thymic States) scale was designed and validated to define mood states dimensionally. The aim of this scale is to assess activation/inhibition levels in all bipolar mood episodes with a single tool, and to determine whether a clinical description in terms of activation/inhibition can help better define bipolar states with which both manic and depressive symptoms are associated.

Using this scale, it has been shown previously that activation and emotional hyperreactivity (feeling emotions with a higher intensity than usual) are associated with mixed and manic states [5]. Furthermore, three clusters of activation/inhibition levels identified with the MAThyS scale were associated with bipolar depression, manic states, and mixed states correspondingly [5]. The scale also permitted the identification of two types of depression according to the level of activation/inhibition displayed: pure depression, characterized by global inhibition and emotional hyporeactivity, and depression with mixed features, characterized by mild activation and emotional hyperreactivity [6].

The main objective of this analysis was to further assess the psychometric properties of the MAThyS scale, especially the distribution and evolution of the total score in different bipolar subgroups in a 6-month, open-label, single-arm, flexible-dose, multicenter clinical trial.

## Methods

Participants

This open-label study was conducted in 14 French centres from November 2005 to May 2008. The study was approved by the Ethics Committee “*Comité de Protection des personnes Sud-Mediterranee II”* (Marseille, France) and conducted according to applicable laws and regulations, Good Clinical Practice (as defined by the International Conference on Harmonisation), and the Declaration of Helsinki. The study was assigned the ClinicalTrials.gov identifier NCT#002592722. Subjects included were inpatients and outpatients with an adult bipolar disorder diagnosis and currently in acute mood episode (manic, hypomanic, mixed, or depressive) according to the Diagnostic and Statistical Manual of Mental Disorders, fourth edition, text revision (DSM-IV-TR) criteria confirmed by module D of the Structured Clinical Interview for the Diagnostic and Statistical Manual of Mental Disorders. Pregnant or breastfeeding women were excluded, as were patients with an acute, serious, or unstable medical condition [7] or a current or lifetime comorbid DSM-IV-TR Axis I or II diagnosis which could interfere with the evaluations also. Patients at risk of suicide (according to the investigator’s opinion and 17-item Hamilton Depression Rating Scale (HAMD-17) item 3 Suicide ≥3 were also excluded. Patients signed and dated the informed consent document before entering the study. The study was approved by ethical review boards.

### Interventions

The screening period (Study Period I) of 0 to 8 days was followed by the 6-week acute phase (Study Period II) with one visit per week for the first 3 weeks (Visits 1 to 5). The maintenance phase (Study Period III) consisted of an additional 18 weeks of follow-up with one visit, which occurred 6 weeks after the beginning of this period (Visits 5 to 7). “Endpoint” refers to the last non-missing observation in Study Period II (acute phase endpoint) or III (overall endpoint) (Additional file 1: Figure A). All patients received oral olanzapine in tablets for the treatment of acute episodes according to their diagnoses and clinical states: an initial daily dose of 15 mg/day for manic and mixed state, 10 mg/day for hypomanic, or 5 mg/day for depressive state, and then adjusted to 5–20 mg/day if clinically indicated. Olanzapine as monotherapy or in combination with lithium or valproate is indicated in the treatment of acute manic or mixed episodes and in delaying the time to and rate of relapse of manic, mixed, or depressive episodes in adult patients with bipolar disorder. Since 2003, in some countries olanzapine is indicated in association with fluoxetine in bipolar depression (Symbyax®). In this study, olanzapine monotherapy was explored in a group of bipolar depressive patients, off-label in France [8]. Concurrent use of benzodiazepine not exceeding 4 mg/day of lorazepam-equivalent, antipsychotics with a sedative action not exceeding 75 mg/day of levomepromazine-equivalent or 100 mg/day of cyamemazine-equivalent were allowed.

Patients were interviewed by a trained psychiatrist at 0, 1, 2, 6, 12 and 24 weeks with the following assessments: MAThyS scale (Additional file 2), HAMD-17, Young Mania Rating Scale (YMRS), and Hamilton Anxiety Scale (HAMA).

The MAThyS scale is a 20-item self-rated visual analogue scale to be used with the assistance of a clinician. This scale was designed a priori, with five quantitative dimensions, which vary from inhibition to activation. The goal was to generate a total score indicative of the overall level of inhibition/activation. Thus, classic dimensions, such as cognition, motivation, psychomotor agitation or retardation, and sensory perception were assessed quantitatively (i.e., racing thoughts or subjectively feeling that their thoughts occur slower, physical agitation or retardation, and increase or decrease in sensory perception). We applied a similar concept to evaluate emotion, focusing only on the quantitative aspect (i.e., whether the patient felt emotion with normal intensity, greater intensity, or less intensity). The patient had to indicate how he felt during the last week for each item by marking a vertical line on a 10-cm horizontal line representing a complete spectrum from inhibition to activation, with the middle of the line representing the usual state. A score of 0 indicates inhibition, whereas a score of 10 indicates excitation for the evaluated item. Items are measured in centimeters from the left, except for the reversed items 5, 6, 7, 8, 9, 10, 17, and 18. The total score can be quoted from 0 to 200 since it is obtained by summing the items’ scores.

### Outcomes

The primary objective of this study was the validation of the MAThyS scale in a population of inpatients and outpatients aged ≥18 years, suffering from bipolar disorder I or II, and currently in an acute episode. This study assessed the psychometric properties of the tool: internal consistency; dimensional structure; responsiveness to the clinical intervention through MAThyS score change and effect size at 6 weeks (acute endpoint) and 24 weeks (overall endpoint); and concurrent validity (correlation with YMRS, HAMD-17, and HAMA).

This article focuses on the primary objective, the secondary objectives of the study were the assessment of efficacy and safety of olanzapine, as all included patients received oral olanzapine, and have been discussed elsewhere [7,9].

### Sample size

The sample size was calculated to allow sufficiently accurate standard errors for the estimated parameters of the factor analysis. As the distribution of the variables and their covariance matrix were unknown, the sample size was determined following the recommendation discussed in the literature [10]. Slow recruitment led to a revision of the sample size calculation to 140 patients which would still allow sufficient statistical power for the primary analysis. This new calculation could have jeopardized (in theory) the precision of the coefficients of the factorial analysis; however, no issues related to precision occurred.

A target of 50 depressive patients and 30 in each of the other subgroup (manic, mixed or hypomanic) was proposed to take into consideration the hypothesized distribution of the activation/inhibition process.

### Statistical analysis

The primary analysis and efficacy analyses were conducted in an intent-to-treat population. All the analyses were done using SAS® v.8.02.

#### MAThyS validation

Pearson correlation coefficients between all pairs of items of the MAThyS scale at baseline were computed.

A principal component analysis was performed with the MAThyS scale items at baseline to identify the structure of the scale (optimal number of factors). A scree plot of eigenvalues was provided. The optimal number of factors was defined according to different approaches (scree plot, Kaiser’s eigenvalues-greater-than-1 rule). Percentages of variance explained by each factor and cumulative percentages were described. A maximum likelihood factor analysis on the MAThyS scale items at baseline was used to assess the varimax rotation loadings.

MAThyS scale’s total score and subscores at baseline were described in the overall population and in each bipolar disorder subgroup. Bipolar disorder subgroup scores were compared using a Kruskal-Wallis test. Subgroups were compared 2 by 2 (Wilcoxon test). For the 2 by 2 comparisons, the significance level was adjusted for multiple comparisons and set to 0.0083 (Bonferroni’s adjustment of the p-value). Internal consistency was assessed for each dimension of the MAThyS scale at baseline using Cronbach’s coefficient alpha and their 95% confidence intervals (CI) computed using the bootstrap approach. Concurrent validity was assessed: correlations between MAThyS scale scores and HAMD-17, HAMA, and YMRS were provided at each time point and at the endpoint. Changes from baseline to acute and overall endpoints and their 95% CI were described for the MAThyS scale, HAMD-17, HAMA, and YMRS scores in the overall population and in each subgroup.

## Results

Investigators screened 150 patients in 14 centers; 141 patients were included: 36 manic, 31 hypomanic, 26 mixed, and 48 depressive patients. Nine patients were excluded due to ineligibility criteria (n = 5), an adverse event before having received olanzapine (n = 1), and refusal (n = 3). Of these 141 included patients, 101 completed the acute phase and 93 the maintenance phase. MAThyS’s factor analysis was performed on the 139 included patients who completed the scale (1 patient did not complete the scale at baseline and another only partially).

The olanzapine mean dose in this study was 10.61 mg/day (±4.21) for the total sample: 14.90 mg/day (±2.89) for the manic episode group, 10.05 mg/day (±2.35) for hypomanic, 12.25 mg/day (±3.78) for mixed episode, and 6.87 mg/day (±2.31) for the depressive episode group.

The patients’ baseline characteristics have been presented in Table 1.

**Table 1:** Baseline characteristics of patients included in MATHYS clinical trial in acute mood episode

	**Manic n = 36**	**Hypomanic n = 31**	**Mixed n = 26**	**Depressive n = 48**	**Global N = 141**
**Sociodemographic characteristics**					
Sex, female (n,%)	19 (52.8)	21 (67.7)	15 (57.7)	25 (52.1)	80 (56.7)
Age (mean, SD)	46.4 (15.3)	45.8 (11.7)	46.6 (12.9)	44.4 (12.0)	45.6 (12.9)
BMI (mean, SD)	24.8 (4.5)	24.7 (5.0)	26.9 (7.8)	24.5 (3.7)	25.1 (5.2)
Status, inpatient (n,%)	26 (72.2)	12 (38.7)	10 (38.5)	24 (50.0)	72 (51.1)
**Psychiatric history**					
Bipolar type (n,%)					
I	36 (100.0)	14 (45.2)	26 (100.0)	31 (64.6)	107 (75.9)
II	0 (0.0)	17 (54.8)	2 (7.7)	17 (35.4)	36 (25.5)
Age at onset (mean, SD)	30.6 (13.9)	34.0 (13.8)	27.4 (10.8)	29.2 (10.4)	30.3 (12.3)
Duration of illness in years (mean, SD)	16.9 (13.9)	12.5 (9.5)	19.3 (14.5)	15.7 (11.6)	15.9 (12.5)
Number of episodes lifetime (mean, SD)	7.5 (4.8)	8.3 (7.6)	6.5 (5.9)	7.2 (5.8)	7.4 (6.0)
Duration of current episode in months (mean, SD)	1.0 (1.5)	2.0 (3.2)	1.7 (1.6)	1.5 (1.5)	1.5 (2.0)
Lifetime substance-use disorder (n,%)					
Alcohol dependence	2 (5.6)	1 (3.2)	4 (15.4)	3 (6.3)	10 (7.1)
Alcohol abuse	5 (13.9)	4 (12.9)	6 (23.1)	12 (25.0)	27 (19.1)
Other substances	1 (2.8)	1 (3.2)	0 (0.0)	4 (8.3)	6 (4.3)
Lifetime suicide attempts (n,%)	10 (29.4)	5 (17.9)	10 (40.0)	18 (39.1)	43 (32.4)
Previous olanzapine treatment (n,%)	16 (44.4)	10 (32.3)	7 (26.9)	17 (35.4)	50 (35.5)
**Rating scales**					
MAThyS total score (mean, SD)	142.2 (23.0)	137.7 (23.4)	116.2 (21.4)	75.3 (23.9)	113.20 (37.1)
HAMD-17 total score (mean, SD)	6.5 (4.9)	8.4 (5.3)	12.7 (4.8)	16.1 (7.2)	11.4 (7.0)-
YMRS total score (mean, SD)	24.5 (10.3)	17.8 (4.9)	13.0 (7.7)	2. 8 (3.6)	13.7 (11.0)-
HAMA total score (mean, SD)	7.8 (6.3)	11.9 (7.7)	15.5 (7.2)	15.1 (7.4)	12.6 (7.7)

Abbreviations: BMI, Body Mass Index; HAMA, Hamilton Anxiety Scale; HAMD-17, 17-item Hamilton Depression Scale; MAThyS, Multidimensional Assessment of Thymic State; SD, Standard Deviation; YMRS, Young Mania Rating Scale.

### MAThyS scale properties

The inter-item correlations showed satisfactory coefficients (above 0.2), except for item 5 for which correlations with all the other items were below or equal to 0.2. The hypothesized distribution of scores according to the subgroups was observed for baseline MAThyS scale’s total scores and scores by item: manic patients had the greatest mean score, followed by hypomanic, mixed, and depressive patients, respectively.

For all items except item 5, manic and hypomanic patients had a mean score higher than 50/100 (50 corresponds to the middle of the visual scale, considered to be the patient’s usual state; a score higher than 50 corresponds to activation and lower than 50 to inhibition), whereas depressive patients scored less than 50. Mixed patients had a score distribution with the same trend as manic and hypomanic patients, except for some items (14, 15, 16, and 17) where they scored less than 50. Total scores were coherently distributed (high for manic, hypomanic, and mixed episodes; low for depressive episodes).The comparison of MAThyS total score by subgroups of episode at baseline showed a significant difference between each group 2 by 2 (adjusted p < 0.0083), except between hypomanic and manic subgroups (Table 1).

### Principal component and factor analysis

According to the scree plot of eigenvalues, a 2-factor structure best fits the data (Additional file 3: Figure B). A rotated factorial analysis provided the item distribution shown in Table 2. The first factor was constituted by the following items, according to priority: 15, 16, 11, 14, 17, 4, 19, 13, 2, 20, 10, and1. It seems to cover mostly the concept of activation/inhibition. It explained most of the variance (74%).

**Table 2:** Rotated factorial analysis: factor pattern*

**Item - Key word**	**Charge on factor 1**	**Charge on factor 2**
Item 1 – Color	0.47	0.36
Item 2 – Tonus	0.61	0.32
Item 3 – Anesthesia	0.20	0.72
Item 4 – Inhibition	0.67	0.34
Item 6 – Sensible	0.23	0.43
Item 7 – Mood	0.10	0.81
Item 8 – Music	0.31	0.61
Item 9 - Brady/tachypsychia	0.25	0.51
Item 10 – Reactivity	0.47	0.47
Item 11 – Energy	0.80	0.26
Item 12 – Thoughts	0.42	0.50
Item 13 – Food	0.64	0.31
Item 14 – Communication	0.74	0.17
Item 15 – Motivation	0.84	0.21
Item 16 – Interest	0.81	0.21
Item 17 – Decisions	0.68	0.21
Item 18 – Emotions	0.20	0.74
Item 19 – Movements	0.66	0.28
Item 20 – Odors	0.51	0.46

* Item 5 excluded from the analysis.

The second factor was constituted by items 7, 18, 3, 8, 9, 12, and 6. It seemed to cover most of the emotional part. It explained 13% of the variance.

### Psychometric properties

Internal consistency measured by Cronbach’s criteria was excellent (0.93; 95% CI [0.92; 0.95]). External validity was good; there were baseline correlations of MAThyS scale score with YMRS (0.72) and HAMD-17 (−0.43) scores (Table 3). Correlation with HAMA score (−0.19) was low and thus not in favor of an overlap of anxiety and the dimensions assessed by the MAThyS scale (Table 3).Correlation between MAThyS scale’s total score, HAMD-17, YMRS, and HAMA scores remained significant at each time point (Table 4). The magnitude of MAThyS total score change over time was satisfying with an effect size of −0.3 at Weeks 6 and 24. The change direction is consistent with other scales’ improvement (Table 4).

**Table 3:** Pearson correlations between MAThyS total score and YMRS, HAMD-17, and HAMA at each time point

**Time point**	**Correlation between MAThyS (without item 5) and …:**		
	**YMRS**	**HAMD-17**	**HAM-A**
Baseline (n = 138)	0.72*	−0.43*	−0.19*
Acute endpoint (n = 131)	0.43*	−0.42*	−0.36*
Endpoint (n = 139)	0.46*	−0.45*	−0.38*

* p value <0.05.

Abbreviations: HAMA, Hamilton Anxiety Scale; HAMD-17, 17-item Hamilton Depression Scale; MAThyS, Multidimensional Assessment of Thymic State; YMRS, Young Mania Rating Scale.

**Table 4:** Scales scores evolutions between baseline to acute endpoint (Week 6) and overall endpoint (Week 24)

	**Manic n = 36**		**Hypomanic n = 31**		**Mixed n = 26**		**Depressive n = 47**	
Scale; mean, total score (SD) / 95%CI	Change baseline-acute endpoint	Change baseline-endpoint	Change baseline-acute endpoint	Change baseline-endpoint	Change baseline-acute endpoint	Change baseline-endpoint	Change baseline-acute endpoint	Change baseline-endpoint
**MAThyS without item 5**	**−24.8 (29,2) [−35.7; -13.9]**	**−25.6 (25.6) [−34.5; -16.7]**	**−33.0 (31.7) [−44.6; -21.3]**	**−34.4 (34.0) [−46.8; -21.9]**	**−12.9 (27,0) [−24.2; -1.5]**	**−26.3 (42.0) [−43.6; -9.0]**	**18.0 (26,9) [9.8; 26.1]**	**18.2 (33.3) [8.4; 28.0]**
**Factor 1**	−21.7 (32.5) [−33.6;-9.8]	−24.5 (28.8) [−34.4;-14.6]	−35.6 (36.6) [−49.0;-22.2]	−34.4 (42.7) [−50.1;-18.7]	−6.1 (32.8) [−19.9;7.8]	−21.8 (44.7) [−40.2; -3.3]	25.3 (29.1) [16.4;34.1]	26.9 (34.2) [16.8;36.9]
**Factor 2**	−27.2 (34.6) [−40.1;-14.3]	−26.2 (30.0) [−36.6;-15.7]	−28.4 (33.8) [−40.8;-16.0]	−34.3 (30.1) [−45.3;-23.3]	−24.5 (29.9) [−37.2;-11.9]	−35.7 (45.8) [−54.6;-16.8]	4.9 (32.0) [−4.7;14.6]	3.3 (38.3) [−8.0;14.6]
**HAMA**	−2.7 (4.9) [−4.5; -0.9]	−2.3 (4.8)[ −3.9; -0.6]	−5.5 (5.9) [−7.7; -3.3]	−5.7 (7.4) [−8.3; -3.0]	−7.3 (6.3) [−9.9; -4.7]	−7.6 (7.9) [−10.8; -4.4]	−6.8 (6.6) [−8.7; -4.8]	−6.8 (7.2) [−8.9; -4.7]
**HAMD-17**	−1.8 (4.6) [−3.5; -0.1]	−1.7 (5.0) [−3.5; 0.0]	−3.1 (4.6) [−4.9; -1.4]	−3.2 (6.4) [−5.6; -0.8]	−5.6 (6.6) [−8.3; -2.9]	−5.5 (6.7) [−8.2; -2.8]	−8.5 (6.2) [−10.4; -6.6]	−8.0 (7.2) [−10.1; -5.9]
**YMRS**	−13.7 (9.4) [−17.1; -10.2]	−14.0 (10.9) [−17.8; -10.2]	−13.1 (6.0) [−15.3; -10.9]	−14.5 (7.4) [−17.2; -11.8]	−9. 7 (7.9) [−13.0; -6.3]	−10.6 (8.0) [−13.9; -7.3]	−0.9 (3.5) [−2.0; 0.2]	−0.8 (3.2) [−1.8; 0.2]

Abbreviations: HAMA, Hamilton Anxiety Scale; HAMD-17, 17-item Hamilton Depression Scale; MAThys, Multidimensional Assessment of Thymic State; SD, standard deviation; YMRS, Young Mania Rating Scale.

### MAThyS scale score

**Figure 1:**
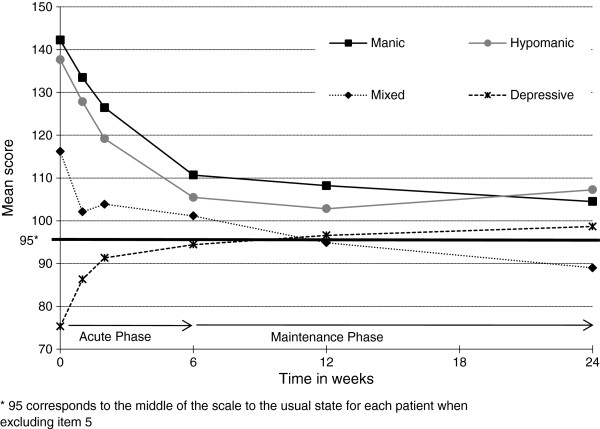
MAThyS total score.

The MAThyS scale score was distributed coherently during this study through each subgroup (score decreased for manic, hypomanic, and mixed patients while it increased for depressive patients) and improved in each subgroup except for mixed patients; most of the improvement was seen during the acute phase (Figure 1). Although no statistical comparison was performed, improvement observed in the scales assessing mood was consistent with the type of episode observed. Manic, hypomanic, and mixed patients had a clinically meaningful decrease in the YMRS for both endpoints and a clinically meaningful decrease of MAThyS at 6 weeks and at 24 weeks (Table 4). Mixed and depressive patients had a meaningful decrease in the HAMD-17 for both endpoints and a clinically meaningful change in the MAThyS’ total score at 6 and 24 weeks (increase for depressive patients and decrease for mixed patients).

## Discussion

The scale had good psychometric properties, did not overlap with anxiety assessments, and showed results in favour of sensitivity to change. At baseline, the total score was distributed coherently according to the expected level of activation in the different mood episodes.

This validation study had several strengths. Firstly, it benefited from the multicenter design and monitoring of an industry-sponsored clinical trial in terms of included study population, investigators’ training, and control of the intervention (olanzapine treatment and concomitant medications). Secondly, the longitudinal nature of the trial allowed the measurement of changes over the time. Thirdly, it included a broad spectrum of mood episodes to observe the difference in the distribution of the inhibition/activation process.

Several results need to be highlighted. In this sample, we observed a 2-dimension factorial structure. One of them was dominant and seemed to cover a concept that might be labeled “level of activation”. The level of activation includes dimensions such as the subjective impression of speed of cognitive process, level of motivation, psychomotor agitation and sensory perception. The second dimension may be labeled “emotional component”. The differences between these findings and the previous five dimensions observed [3] may be explained by differing populations and study designs. Interestingly, the five previously described dimensions also fit the data well in a post hoc confirmatory analysis (data not showed). From a practical point of view, the relevance of a total score in discriminating between some difficult-to-diagnose mood episodes (such as mixed episodes) and following up their evolution is important for clinicians and clinical practice.

In this study, the total MAThyS scale’s score was distributed as hypothesized between the different subgroups: manic, hypomanic, and mixed subgroups had higher scores relative to the mean (100), corresponding to a global activation process; while depressive episodes had lower scores relative to the mean with a global inhibition process. Additional features of the MAThyS total score that add to its clinical value are that it did not overlap with anxiety (which may have been a confounding factor) and that it changed over time consistently, compared to other scales, in all patients parallel to clinical improvement in each subgroup. These data suggest that the use of a total MAThyS scale score is a legitimate means of discriminating between the different mood episodes in bipolar disorder, especially the difficult differential diagnosis between a mixed episode (with activation) and depression (with inhibition) and may be a possible additional marker for following the clinical course of these patients.

Difficulty with differential diagnoses may lead to the inadequate prescription of antidepressant monotherapy, which may worsen the prognosis. Interestingly, in mixed states, the total score of MAThyS was specifically associated with a continuous modification of the score until 24 weeks, while it stabilized in the other subgroups after 6 weeks. In this case, the distribution of the items in both the hyperreactivity and hyporeactivity sides makes the interpretation of this evolution quite complex. Whereas mixed states scored consistently higher on the activation items, inhibition was observed on a set of 5 items, which seems to be associated with executive function and motivational process (items 9:speed of mind, 14:communication, 15:motivation, 16:interest, and 17:decision making). Different explanations that are not mutually exclusive may be proposed: a continuous improvement, a switch to inhibition, or the known longer delay to remission of mixed states [10].

Our study presented some limitations that should be taken into account when interpreting the results. While in this sample the improvement of depressive patients treated with olanzapine monotherapy was satisfactory [7], olanzapine monotherapy failed to demonstrate sufficient efficacy in a specifically designed clinical trial [11]. Further studies with larger samples should assess the impact of registered treatments of depression (antidepressants, other atypical antipsychotics, etc.) on the inhibition/activation process.

A slower than anticipated recruitment led to a revision of the sample size calculation which still allowed for a sufficient statistical power for the primary analysis. The difficulty in enrolling the initially planned number of patients is likely linked to the fact that the validation of the MAThyS was performed during a clinical trial including *per se* some constraints (inclusion/exclusion criteria, follow-up visits, etc.), and was not due to the acceptability of the scale which has been found to have good acceptability independent of severity of the episode [5].

One item (item 5: distractibility and attention) had to be excluded from the analysis as it showed low correlations with other items. One explanation for the instability of item 5 is that the language used for this item in the French version was ambiguous. It appeared to be difficult to assess by patients because its extreme points (distractibility for mania and loss of attention for depression) were difficult to differentiate clinically and are potentially disturbed in both depression and mania. A reformulation of this item is necessary. The interpretation of our results should thus be limited to the MAThyS scale restricted to 19 items, but the impact of this exclusion should be moderate as the weight of this item was low in our analysis.

Another important limitation is that there is no control group in this study to assess the sensitivity to change.

## Conclusions

Assessment of the inhibition/activation process is a tool for diagnosing bipolar symptomatology. Not only is this process highly correlated in acute states with classical thymic categorical evaluation, but it can also allow a more accurate discrimination between mixed states and depression.

This study suggests that the MAThyS scale may be a useful dimensional tool, along with categorical tools, to discriminate and follow the different bipolar mood episodes, particularly for mixed states which are usually severe, underdiagnosed, and mistreated.

## Competing interests

Chantal Henry, Christophe Lançon, Marcel Zins-Ritter, and Bruno Falissard have received honoraria from Lilly for consultancy work and/or to be an investigator. Elena Perrin, Hélène Sapin, and Stephanie Gerard are currently working for Eli Lilly and Company. Amandine Luquiens and Michael Lukasiewicz are former Lilly France employees but were Lilly employees when the manuscript was drafted.

## Authors’ contributions

CH was the expert who has built the scale and has participated in the design of the objectives; she was an investigator, participated to data interpretation and reviewed the manuscript. HS performed the statistical analysis, participated to data interpretation and reviewed the manuscript. ML, AL and SG have interpreted the data and drafted the manuscript. CL and MZ-R were investigators and have reviewed the manuscript. BF and EP participated to data interpretation and reviewed the manuscript. All authors read and approved the final manuscript.

## Pre-publication history

The pre-publication history for this paper can be accessed here:

http://www.biomedcentral.com/1471-244X/13/79/prepub

## Supplementary Material

Additional file 1: Figure A Study Design. Click here for file

Additional file 2 Additional document – The MAThyS (Multidimensional Assessment of Thymic States) scale in French. Click here for file

Additional file 3: Figure B Principal component analysis: scree plot of eigenvalues. Click here for file
