# Supplementary material for: Inhibition/activation in bipolar disorder: validation of the Multidimensional Assessment of Thymic States scale (MAThyS)
Source: BMC Psychiatry. 2013 Mar 13;13:79. doi: 10.1186/1471-244X-13-79 (PMC3600043; doi:10.1186/1471-244X-13-79)
Supplement: Additional file 1: Figure A — Study Design. [file 1471-244X-13-79-S1.docx]

**Online additional documents**

**Figure A: Study design**

**Study Period I**

**Study Period II**

**Study Period III**

Visit 0

Visit 5

Visit 6

Visit 7

Scre

e

ning

Period

Maintenance Phase

18 weeks

O

lanzapine flexible dose

6

weeks

12 weeks

0

-

8 days

All

Patient

s

Acute

Treatment

Phase

6 weeks

Visit

2

V

isit

3

Visit

4

1 w

ee

k

1 w

ee

k

1

wee

k

3 w

ee

ks

Visit 1

Total = 24 weeks
