# Supplementary material for: Inhibition/activation in bipolar disorder: validation of the Multidimensional Assessment of Thymic States scale (MAThyS)
Source: BMC Psychiatry. 2013 Mar 13;13:79. doi: 10.1186/1471-244X-13-79 (PMC3600043; doi:10.1186/1471-244X-13-79)
Supplement: Additional file 2 — Additional document – The MAThyS (Multidimensional Assessment of Thymic States) scale in French. [file 1471-244X-13-79-S2.docx]

**Additional document – The MAThyS (Multidimensional Assessment of Thymic States) scale in French**

Cette échelle a pour but **d'évaluer votre état** au cours de la **dernière semaine**. Pour chaque item, indiquez par un trait où vous pensez vous situer en fonction des deux propositions sachant que le centre du trait représente votre état habituel

| 1- Je suis moins sensible que d’habitude aux couleurs |  | Je suis plus sensible que d’habitude aux couleurs |
| --- | --- | --- |
| 2- Je manque de tonus |  | J’ai une tension musculaire importante |
| 3- J'ai l'impression d'être anesthésié(e) sur le plan des émotions |  | J'ai parfois le sentiment de perdre le contrôle de mes émotions |
| 4- Je suis replié(e) sur moi |  | Je suis désinhibé(e) |
| 5- Je suis facilement distrait(e), la moindre chose me fait perdre mon attention |  | Je ne suis pas attentif à mon environnement |
| 6- Je suis plus sensible que d’habitude au toucher |  | Je suis moins sensible que d’habitude au toucher |
| 7- J’ai l’impression que mon humeur varie beaucoup en fonction de mon environnement |  | Mon humeur est monotone et peu changeante |
| 8- Je suis particulièrement sensible à la musique |  | Je suis plus indifférent que d'habitude à la musique |
| 9- Mon cerveau ne s’arrête jamais |  | Mon cerveau fonctionne au ralenti |
| 10- Je suis plus réactif (ve) à mon environnement |  | Je suis moins réactif à mon environnement |
| 12- J’ai le sentiment que mes pensées sont ralenties |  | J’ai le sentiment que mes idées défilent dans ma tête |
| 13- Je trouve la nourriture sans goût |  | Je recherche les plaisirs gastronomiques car j'en apprécie davantage les saveurs |
| 14- J’ai moins envie de communiquer avec les autres |  | J'ai plus envie de communiquer avec les autres |
| 15- Je manque de motivation pour aller de l'avant |  | Je multiplie les projets nouveaux |
| 16- Ma perte d’intérêt pour mon environnement m’empêche de gérer le quotidien. |  | J’ai envie de faire plus de choses que d’habitude |
| 17-Je prends les décisions de manière plus rapide que d’habitude. |  | J’ai plus de difficultés que d’habitude à prendre des décisions |
| 18- Je ressens les émotions de manière très intense. |  | Mes émotions sont atténuées |
| 19- Je suis ralenti(e) dans mes mouvements. |  | Je suis physiquement agité(e) |
| 20- J’ai l’impression d'être moins sensible aux odeurs que d’habitude. |  | J’ai l’impression d’être plus sensible aux odeurs que d’habitude |

| **Score :** |  |  |  |
| --- | --- | --- | --- |

Au cours de cette même période, précisez quels types d’émotions vous avez ressentis, en précisant leur fréquence :

**Tristesse**  Jamais  Occasionnellement  Souvent  Très souvent  Constamment

**Joie**  Jamais  Occasionnellement  Souvent  Très souvent  Constamment

**Irritabilité**  Jamais  Occasionnellement  Souvent  Très souvent  Constamment

**Panique**  Jamais  Occasionnellement  Souvent  Très souvent  Constamment

**Anxiété**  Jamais  Occasionnellement  Souvent  Très souvent  Constamment

**Colère**  Jamais  Occasionnellement  Souvent  Très souvent  Constamment

**Exaltation**  Jamais  Occasionnellement  Souvent  Très souvent  Constamment
