# Supplementary figures and images for: Inhibition/activation in bipolar disorder: validation of the Multidimensional Assessment of Thymic States scale (MAThyS)
Source: BMC Psychiatry. 2013 Mar 13;13:79. doi: 10.1186/1471-244X-13-79 (PMC3600043; doi:10.1186/1471-244X-13-79)

**Figure B :Principal component analysis: scree plot of eigenvalues**


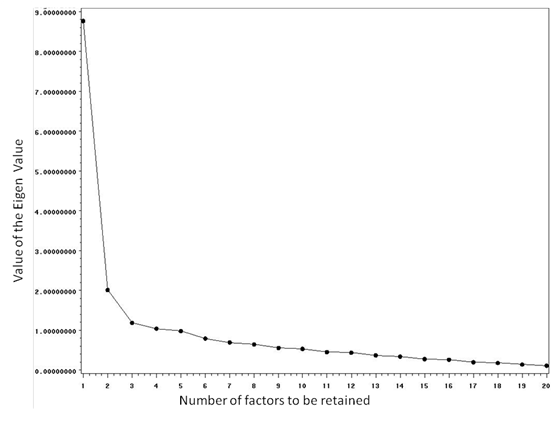

Supplement: Additional file 3: Figure B — Principal component analysis: scree plot of eigenvalues. [file 1471-244X-13-79-S3.docx]
